# Strategies and tools to learn from work that goes well within healthcare patient safety practices: a mixed methods systematic review

**DOI:** 10.1186/s12913-025-12680-2

**Published:** 2025-04-14

**Authors:** Gørill Birkeli, Anne Karin Lindahl, Åse Marit Hammersbøen, Ellen Catharina Tveter Deilkås, Randi Ballangrud

**Affiliations:** 1 Division of Surgery, Akershus University Hospital, Sykehusveien 25, Lørenskog, 1478 Norway; 2 Institute of Health and Society, Department of Health Management and Health Economics, Faculty of Medicine, University of Oslo, Oslo, Norway; 3 Department of Research Support, Akershus University Hospital, Lørenskog, Norway; 4 Health Services Research Unit, Akershus University Hospital, Lørenskog, Norway; 5 Department of Quality Improvement and Patient Safety, Norwegian Directorate of Health, Oslo, Norway; 6 Faculty of Medicine and Health Sciences, Department of Health Sciences, Norwegian University of Science and Technology, Gjøvik, Norway

**Keywords:** Systematic review, Healthcare, Interventions, Safety-II, Learning, Quality improvement, Reporting

## Abstract

**Background:**

Safety-II is a new approach to patient safety that is characterised by learning from work that goes well, including learning from success and work-as-done. Practical tools to facilitate this learning are starting to emerge within healthcare patient safety practices. In absence of a systematic review of such learning tools, the aim of the study was to provide an overview of strategies and tools for healthcare professionals to learn from work that goes well in healthcare patient safety practices.

**Methods:**

Registered in advance in PROSPERO, this systematic review has followed the PRISMA 2020 checklist. We searched eight databases in February 2023: Medline, Cinahl, Embase, PsycInfo, Cochrane Central, Web of Science, Scopus, and Google Scholar. Articles describing the development, implementation or evaluation of tools were included if they were (1) quantitative, qualitative, mixed-methods or white papers/commentaries (non-empirical), (2) available in English or Scandinavian language, (3) published between 2000 and February 2023, (4) developed or implemented in healthcare practices, (5) detailed in description and (6) preferably peer-reviewed. Articles were excluded if they primarily dealt with students, Functional Resonance Analysis Method (FRAM), appreciative inquiry and positive deviance. Articles were screened against eligibility criteria using Rayyan software. The Mixed Methods Appraisal Tool was used to assess the quality of the articles. The framework for resilience research was used to present and synthesise the results.

**Results:**

Out of 5298 records screened, 126 articles were retrieved for evaluation, and 22 articles were included, describing 16 unique tools. Five tools were not empirically evaluated. Most learning tools were aimed at healthcare professionals in hospitals units (68%), and were generally welcomed by healthcare professionals. Tools intended for learning across the organisation were second most frequent (23%), followed by tools intended for learning between hospitals (9%). Most studies focused on validating the tools’ ability to provide insights into work-as-done, and their effect on staff wellbeing. Few studies focused on patient outcomes.

**Conclusions:**

The review shows a growing number of practical Safety-II tools, which may help understand and learn from the constant adaptations made by healthcare professionals every day to keep patients safe.

**Trial registration:**

PROSPERO: number CRD42022335758.

**Supplementary Information:**

The online version contains supplementary material available at 10.1186/s12913-025-12680-2.

## Background

Patient safety may be understood as a “framework of organised activities (…) that lower risks, reduce the occurrence of avoidable harm, make errors less likely and reduces the impact of harm when it does occur” [1]. Although patient safety is a strategic priority for modern health care, adverse events is still the 14 th leading cause of the global disease burden [2, 3]. Between 3 and 16% of hospitalised patients suffer harm from medical care and this number seems to be stalling [4, 5]. Incident reporting systems are considered cornerstones of the traditional approach to patient safety (Safety-I). Accordingly, healthcare systems have different methods for incident reporting, all of which can identify different types of risks to inform quality improvements and facilitate continuous learning [6]. Unfortunately, underreporting is highly prevalent, and is linked to, among other things, shaming and blaming mentality, insufficient visible measures and inadequate communication about errors [7]. Furthermore, most reporting systems do not facilitate learning and, hence, do not improve patient safety [8, 9]. As the aftermath of errors, healthcare professionals may experience the second victim phenomenon including, amongst other things, burnout and depression [10–13]. The link between working climate and patient safety adds to the limitations of focusing on errors [14].

Safety-II is a new approach to patient safety that is characterised by learning from success [15]. The Safety-II perspective has been met with enthusiasm in healthcare practices because it points out that most times things go well despite changing conditions and should be focused on and learned from [15–17]. Safety-II is based on the grand theory of resilience engineering that describes how human activity “adapts to challenges and changes at different system levels, to maintain high quality care” [15, 18]. Resilience engineering draws on the concepts of complex systems [19]. Healthcare systems can be viewed as non-linear, unpredictable complex systems that constantly require healthcare professionals to adapt to the ever-changing conditions, such as shortcomings of staff, miscommunications, overflow of patients, etc., for keeping patients safe [20]. More protocols to constrain how quality care is achieved are not always helpful, because protocols cannot possibly foresee every interaction that may affect the work. When looking at patient safety through the lens of resilience, the focus is on how work is actually done (WAD) rather than how work is imagined (WAI) when looking at protocols [15]. This can inform the distribution of resources and support healthcare professionals’ ability to adapt to allow successful outcomes to happen more frequently [18].

To visualise and explore WAD, Hollnagel et al. [21] introduced the Functional Resonance Analysis method (FRAM). Subsequently, he introduced the Resilience Analysis Grid to explore the presence of the four key potentials that are proposed to create successful WAD: *anticipating, monitoring, responding* and *learning* [22, 23]. Regarding *learning*, Hollnagel stressed that healthcare professionals should learn from both positive and negative experiences to increase positive outcomes and avoid negative ones. In addition, success is more frequent than failure; therefore, it offers more learning opportunities [24]. Learning from positive experiences is separate from, but related to, positive deviance and appreciative inquiry methodology, which specifically look at exceptionally performing individuals to learn from them and disseminate their behaviour [25–27]. Extensive systematic literature reviews have been performed previously regarding FRAM [28, 29], appreciative inquiry [30] and positive deviance [31, 32]. Therefore, these methods were excluded from this systematic review.

Challenges regarding practical implementation of the Safety-II perspective hamper its adoption in healthcare [33–35]. For instance, which activities the potential of *learning* must encompass, what should be learned, and who should be involved remains unclear [36]. Examples of learning on different scales of time and space have been provided theoretically [36]. However, exactly *how* learning from events that go well can be operationalised in healthcare practices remains another matter. This systematic review focuses on tools or strategies to learn from everyday work that goes well in healthcare, thereby operationalising the Safety-II perspective. This includes learning from success and WAD. In this context, a learning tool or strategy supports organisational learning, i.e., helps produce insights and inventions [1, 37–39]. Hereby referred to as *tools.* We believe that this review can be useful for healthcare professionals and researchers, as it may provide a clearer understanding of the range and usability of published Safety-II learning tools.

The aim of the study was to provide an overview of tools for healthcare professionals to learn from work that goes well in healthcare patient safety practices. The following research questions guided the study:

- Which tools are currently practiced to learn from work that goes well in healthcare?
- Which detailed steps do the tools consist of?
- What are the tools’ outcomes (e.g., feasibility, acceptability, effectiveness)

## Methods

### Study design

A mixed studies systematic review, as described by Pluye and Hong [40] was conducted. This approach allowed the synthesis of deductive and inductive data with diverse designs, thereby facilitating a better understanding of how learning from success is currently operationalised in healthcare practices. The review followed seven stages of a systematic review, which are: (1) formulating a research question, (2) defining eligibility criteria, (3) applying an extensive search strategy, (4) identifying potentially relevant studies, (5) studying selection, (6) appraising the quality, and (7) synthesising the included studies [40].

The Preferred Reporting Items for Systematic Reviews and Meta-Analyses (PRISMA) 2020 guidelines was used to ensure thorough and transparent reporting [41]. The study was registered in the PROSPERO International Register of Systematic Reviews (CRD42022335758), available from https://www.crd.york.ac.uk/PROSPERO/view/CRD42022335758.

### Eligibility criteria

The inclusion and exclusion criteria are outlined in Table 1.

**Table 1:** Inclusion and exclusion criteria

Criterion	Articles’ Characteristics
Inclusion	- Published in English or Scandinavian language in peer-reviewed journals between January 2000 and February 2023 - Described the development, implementation or evaluation of tools to learn from success or work-as-done in healthcare patient safety practices - Mentioned tools designed to be used in simulations or real clinical settings - Described the tool’s development, implementation or evaluation in detail - Peer-reviewed (such articles were preferred); however, if a tool had been described and not been published in peer-reviewed journals, this was highlighted
Exclusion	- Dealt with settings outside the healthcare, such as dental care - Had students as their main target group - Focused on increasing resilience to prevent burnout among healthcare professionals - Dealt with the Functional Resonance Analysis Method (FRAM), appreciative inquiry or positive deviance methods

### Information sources

We searched eight databases: (Medline (Ovid), Cinahl (Ebsco), Embase (Ovid), PsycInfo (Ovid), Cochrane Central (Wiley), Web of Science (Clarivate), Scopus (Elsevier), and Google Scholar) in February 2023.

### Search strategy

The search strategy was developed with the assistance of a senior librarian from a medical library [40]. The search combined keywords from four areas:

1. *Resilience* (e.g. work-as-done, work as imagined, work that went well, excellence, success, appreciative, positive feedback)
2. *Healthcare professionals* (e.g. health care, hospitals, health professionals, nurses, physicians and clinicians)
3. *Learning*
4. *Patient safety* (e.g., safety, harm, risk, quality and improvement)

In addition, the concepts *Safety-II* and *resilience engineering* were searched for independently to ensure the capture of learning tools that included these concepts, as they are new.

The search strategy was adopted to each database, and detailed search strategies is shown in Supplementary file 1.

### Selection process

**Fig. 1:**
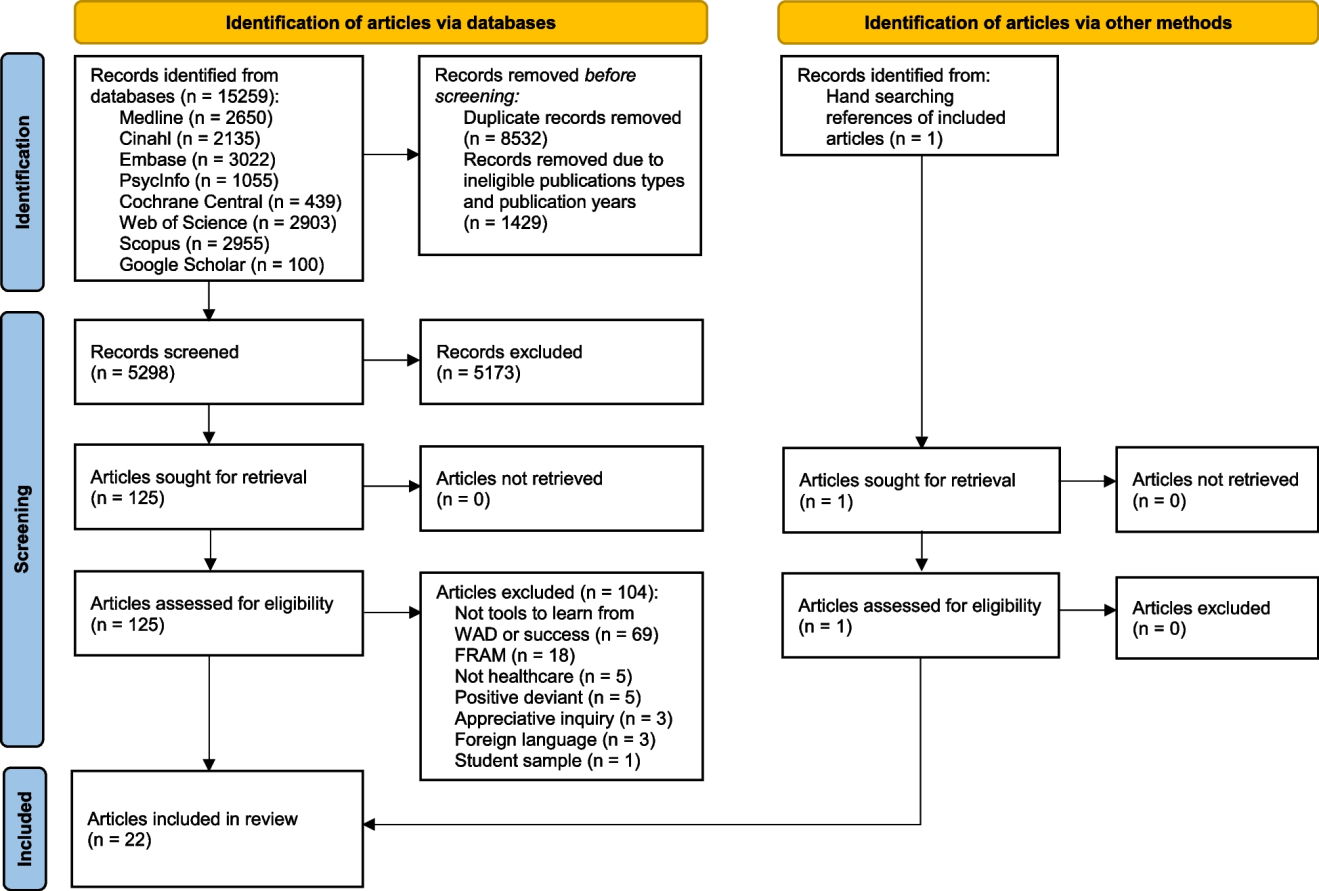
PRISMA 2020 diagram for tools to learn from work-as-done or success in healthcare safety practices

Figure 1 summarises the identification, screening and inclusion process according to the PRISMA 2020 format [41].

#### Identification

Duplicate records from the search were removed by EndNote reference managing tool and Rayyan web-based tool for systematic reviews [42]. Rayyan was used throughout the selection process, thus blinding the reviewers to each other’s decisions.

#### Screening

Two reviewers independently screened the remaining records by titles and abstracts. The senior librarian checked 10 of the rejected records according to the eligibility criteria and agreed that the process worked and that those records should be excluded. Disagreements were resolved by consensus. All reviewers were included in this discussion.

#### Inclusion

A pilot test of the full-text articles was conducted, including the first 10 alphabetically listed articles in Rayyan. These full-text articles were assessed by all reviewers, to ensure that consensus on which one to include was achieved. The reviewers decided that the exclusion-criteria were not quite clear and had to be revised: It was decided to exclude articles that dealt with FRAM, appreciate inquiry and positive deviance, as systematic literature reviews based on these strategies had been done [28, 30, 31]. Then the same two reviewers conducted the full-text review. Reasons for exclusion were documented. Agreement was 95%, and disagreements were resolved by consensus by all reviewers.

### Quality appraisal

Initially, as registered in the PROSPERO protocol, the review team planned to use Joanna Briggs Institute Critical appraisal tools for risk of bias/quality assessment [43]. However, the Mixed Methods Appraisal Tool was deemed more appropriate given that both quantitative and qualitative articles were eligible for inclusion in the review, allowing for assessment with one measure [44–46]. Each type of study has five different criteria, and responses for each criterion can be:’no’ does not meet criteria,’yes’ meets criteria, or’can´t tell’ where appropriate information was not reported. It is advised to present a detailed result of the quality appraisal, and calculation of an overall quality score is discouraged [44]. The quality assessment was carried out independently by GB and RB, and any disagreements were discussed. No other substantial deviations from the registered protocol were made.

### Data extraction

Key details of the included articles were extracted using a form including first author, year, the setting where the tool was introduced, tool’s name, description of tool, and outcome (e.g. feasibility, acceptability, effectiveness) of the tool.

### Data synthesis

The results of the included articles were integrated using a convergent qualitative synthesis that is appropriate to address research questions starting with, e.g. “which or what” [40]. A deductive-inductive thematic analysis was performed, to structure the content into themes and sub-themes [40, 47]. First, the themes were deductively divided into a *situated, structural or systemic level,* informed by framework of Anderson et al.´s for researching resilient performance [36]. Second, sub-themes were inductively created based on the common similarities and differences among the included articles content.

### Risk of including a biased sample

Bias during study selection was minimised through the use of the aforementioned systematic search method [48]. Data analysis was undertaken with an awareness of the potential sources of bias (personal experience, values and beliefs); hence, repeated reflections and discussions among GB, RB, ECTD and AKL were prominent throughout the iterative review of the articles [49]. Bias regarding the assessment of the tools included the following:

- Familiarity bias [50]: GB had previously implemented an LfE tool and was familiar with several of other tools included in this systematic review. However, the rest of the research team did not have this in-depth knowledge, which could mitigate this bias.
- Anchoring bias [51]: The initial organisation of the information presented in each study may influence the convergent qualitative synthesis of the tools. This potential bias was mitigated by applying an objective framework to the analysis of the tools.

## Results

### Study selection

The search strategy identified 5298 records, excluding duplicates, ineligible publication types and publications before 2000. Reference checking resulted in one additional article [52]. A full text screen was performed on 126 articles, of which 104 articles were excluded for the following reasons: not including learning from success or WAD (*n* = 69), including FRAM, positive deviance or appreciate inquiry (*n* = 26), foreign language, not related to healthcare, and the use of student sample (*n* = 9). In total, 22 articles were included in the systematic review (Fig. 1).

### Study characteristics

Sixteen unique tools to operationalise the Safety-II paradigm by learning how work goes well in everyday clinical work, were identified. They were presented in 22 articles describing such a tool. Seventeen of these were empirical research articles from seven high-income countries: United Kingdom *(n* = *9)* [53–61], USA *(n* = *3)* [62–64], and one each from Italy, France, Sweden, Japan and the Netherlands [65–69]. The empirical studies used quantitative methods (*n* = 5), qualitative methods (*n* = 5) and mixed methods (*n* = 7), with mostly descriptive research designs. The results also included 5 non-empirical papers [52, 70–73]. Table 2 presents a detailed description of each study, including author(s), year, setting, tool’s name, description of the tool and outcome of the tool (e.g. feasibility, acceptability, effectiveness). The table also highlights the unique tools (*n* = 16), and the non-empirical papers (*n* = 5). The tools are presented in alphabetical order, and according to the synthesis (see below, and Table 3).

**Table 2:** Summary of the results

Author(s) (Year) [Ref]	Setting (Country of origin)	Tool’s name (Acronym if used)	Tool description	Outcome (e.g., feasibility, acceptability, and effectiveness) of the tool
**SITUATED LEARNING TOOLS: Learning through peer reporting**				
Breinig et al., (2022) [68]	NICU/PICU Children’s hospital (France)	Learning from excellence (LfE)	At least one excellence-report was selected every 3 months by an inter-professional workgroup (3R-team), or the rest of the staff The reports were analysed using the principles of appreciative inquiry. The mission of the 3R-team was to promote excellence, accentuate the positive in various situations, and improve the relationships of the staff	The satisfaction questionnaire (None of these changes were statistically significant.): - 93%: satisfied with the work of the 3R- team - 100%: agreed that it contributed to improving inter-professional communication and relationships - 100% agreed that it contributed to enhancing educational programmes - 88% of respondents agreed that stress in emergency situations had decreased Other outcomes - LfE feasible to implement in a NICU/PICU - Time consuming (3R-team)
Chain et al., (2018) [60]	Surgical ward Children’s hospital (United Kingdom)	Learning from excellence (LfE)	Episode of excellence was observed and reported in a free text form: *Who do you like to nominate? What did they do? How can we learn from their practice?* The reports were collected weekly. The details recorded in Excel documents, and monthly certificates were sent out to the recipient and their supervisor and a copy to the proposer. The feedback recorded on the notes was discussed with the consultants at the monthly surgical morbidity and mortality meeting. Feedback for nursing staff was discussed in meetings with senior staff	Significant positive changes regarding perceived: - The amount and quality of positive feedback - Improved individual and team morale - Potential to improve clinical care - Support for rolling out the project to other parts of the hospital
Jones et al., (2019) [59]	PICU Children’s hospital (United Kingdom)	Learning from excellence (LfE)	Individuals who either achieved gold-standard prescribing practice or administration of timely new antibiotics, received positive feedback through LfE report with a description of what they had achieved and why this was important. Selected LfE reports were followed up with an appreciative interview to enhance positive feedback and gather improvement ideas (10–15 min)	- Antimicrobial consumption was reduced by 6.5% - Meropenem consumption was reduced by 17.6%
Kelly et al., (2016) [61]	PICU Children’s hospital (United Kingdom)	Learning from excellence (LfE)	*Unique tool* Voluntarily reporting of episodes of excellent practice via an internet-based reporting form A small multidisciplinary team of frontline staff review all reports with the submitter and the receiver, using appreciative inquiry methodology Then, creating weekly summaries with learning points, shared through an e-bulletin	Quantitative The survey responses strongly supported the hypothesis that excellence reporting can: - Improve staff morale (93% agree) - Improve quality of care (87% agree) Qualitative Staff perceive that learning from studying excellence is as valuable as reflecting on individual error, and both are more valuable than studying the failure of others
Kletter et al., (2020) [55]	Member of NHS trusts across the UK (United Kingdom)	Learning from excellence (LfE)	Following Kelly et al.´s [61] Learning from excellence program (see description above)	Quantitative - LfE was perceived to have improved morale - Staff was perceived to have a positive response to the intervention - Perceived increased staff retention, change in workplace culture and increased motivation Qualitative LfE was perceived to have positive effects on workplace culture, staff emotions, patient safety and patient experience. No negative effects were noted A programme theory was developed, explaining the following: - How the LfE intervention can potentially impact organisational performance - The mechanisms through which this impact is achieved and the contextual factors that lead to (or away from) LfE´s impact
McGregor et al., (2017) [53]	Acute Medical Unit (United Kingdom)	Save of the month	Multidisciplinary team were asked to identify key factors in why processes worked well in this case. Ideas were generated in an attempt to make that desirable factor happen more reliably The steps to increase patient safety were discussed in daily safety briefings and tested and refined with the full involvement of staff doing the work	- Reduced cardiac arrest rate from 4.3/100 to 1.1/1000 (> 50% reduction) - Learning from success addressed psychological barriers to change by encouraging pride in work and a positive focus
**SITUATED LEARNING TOOLS: Learning through positive debriefing**				
Bartman et al., (2021) [70]	Children´s hospital (United States of America)	Feed Forward The learning tool, Feed Forward, is one of three tools. The two others; the recognition tool (Pause to Predict) and the responding tool (IDEA)	*Unique tool* - Entering learning of unusual actions taken in the patient’s electronic medical record (including situations without an established process, those that run counter to normal workflow, etc.) - After entering this information, a coloured header is visible on the first page of the opened chart, indicating that something unusual has been done proactively - This may help others recognise and respond to similar patient situations in the future	*Non-empirical* A learning tool, Feed Forward, was offered to make things go right more frequently This will be measured by reduction in patient harm, number of adverse events reported in patients with Safety-II plans, number of staff trained in Safety-II tools, number of proactive safety huddles performed and number of safety II plans entered into EHR
Bentley et al., (2021) [64]	Not specified (United States of America)	Debrief it all	*Unique tool* A tool consisting of specific strategies during each debriefing phase (introduction, case summary, take home points), including sample language and phrases, to help learning from both the positive and negative elements of an event The debriefing tool was developed through expert consensus	A tool consisting of specific strategies and sample language and phrases has been developed and offered as a guide Quantitative - The tool added value in debriefings (100% agree) - The tool was readable as phrased and formatted (83% agree) - The questions were understandable (83% agree) - The phrases are clearly linked to concepts indicated in the charts (100% agree) - I would like to include this in future debriefings (100% agree) Qualitative Confirmed the survey responses, e.g.: I will definitely use this again. It helped me expand on the ‘why did it go right’ question I always try to ask
Diaz-Navarro et al., (2021) [71]	Department of perioperative care (United Kingdom)	TALK ©	*Unique tool* - A practical clinical debriefing tool underpinned by values that foster positive communication strategies (positivity, focus on finding solutions, professional communication, step by step) - TALK © consisted of four steps: ◦ Target (What shall we discuss?) ◦ Analysis (What helped or hindred?) ◦ Learning (What can we learn from the experience?) ◦ Key actions (Who will follow up?)	*Non-empirical* Two tables are offered, describing the steps of TALK ®
Dieckmann et al., (2017) [72]	Medical Emergency Team (Denmark)	The learning from success approach in simulation	*Unique tool* Description and examples of learning from good performance in all simulation setting phases (prebriefing, scenario conduct, debriefing), to be used in common everyday situations as a supplement to traditional simulation approaches	*Non-empirical* A table is offered that compares traditional simulation-based education with the learning from success approach, based on selected phases of the simulation
Hegde et al., (2019) [63]	Anaesthesia residents (United States of America)	Resilience Engineering Tool to Improve Patient Safety. Anaesthesia resident-version of the tool (RETIPS-AnRes)	*Unique tool* A lesson-sharing tool developed to operationalise learning from how events go well based on reflections through an electronic-based questionnaire, including -Introduction (purpose of tool) -Case selection (examples that relate to resilience) -Detailed narrative (description of the case in detail) Checkboxes with: - What went right? - Challenges and concerns - Resources - Area of practice	The implementation validated the design in that the responses were aligned with the purpose of the tool, which was to learn about how things go well in everyday clinical work
Verhagen et al., (2020) [69]	Surgical team (The Netherlands)	Quality assessment meetings (QAM)	*Unique tool* A QAM serves as a tool to identify how adapting behaviour led to success despite challenging conditions so that this resilient performance can be supported. It consists of a chronological order of five topics: 1. Inpatient cases (20 min) 2. Severe adverse events (10 min) 3. Patients planned for surgery in the upcoming week (10 min), 4. Operating room timetable (5 min) 5. Literature review (10 min)	Quantitative - QAM is useful as a means to trigger reflection on one´s own decisions and performance (60.6% agreed) - The new format benefits completion of administrative work (73.7% agreed) - Addressing logistic issues for upcoming surgeries (73.6% agreed) Qualitative - Focus on the entire clinical course rather than complications - Evaluation of ‘both sides of the same coin’ - A representation of the ratio between successful and unsuccessful outcomes - Even cases with a better-than-anticipated clinical course pass-in review - Discussing cases with successful outcomes despite suboptimal care
**SITUATED LEARNING TOOLS: Learning through safety-huddles**				
Hollnagel (2020) [52]	Not specified (Denmark)	The Resilient Performance Enhancement Toolkit (RPET)	*Unique tool* RPET is a learning tool to pay attention to and learn from work-as-done. RPET should take place every day, or at least every week, in safety huddles. Subjects for discussion: How did they recognise changes to a situation and how did they handle this? What happens when the same adjustments are made under different conditions? To support continuous learning, it is necessary to track progress in a continuous calendar. Each day is marked with colour codes ranging from not yet discussed to a lesson learnt	*Non-empirical* The RPET is described and offered as a tool to learn from work that goes well
Wahl et al., (2022) [65]	NICU, Children´s hospital (Sweden)	Green Line	*Unique tool* About 5–10 min long reflections in the afternoon with healthcare professionals in the ward Open questions with follow-up questions. For example: How have you succeeded today? How did you manage that? A monthly summary is written of the number of participants, profession, colour classification and which potentials the conversation could be classified within	Quantitative - For most comparisons, no differences were found in safety culture before and after implementing Safety-II safety huddles Qualitative - There were many examples in the safety huddles regarding learning and responding, fewer from anticipating and only one example from monitor - It is difficult to introduce reflections based on the learning from everything that happens, including when things go well into patient safety huddles
Hyman (2016) [73]	Not specified (United States of America)	Success Tree Analysis (STA)	*Unique tool* STA can serve as a tool to identify a good thing you want to happen and then, in turn, identify the next layer of things that have to happen, etc. It can focus improvement efforts on becoming better than on no longer being bad	*Non-empirical* Figures are offered as a guide to perform success tree analysis
**ORGANISATIONAL LEARNING TOOLS: Learning through incident reporting systems**				
Abe et al., (2022) [66]	Yokohama City University Medical Center (Japan)	Extracting Safety-II Factors from an Incident Reporting System by Text Analysis	*Unique tool* Free-text data from the electronic incident reporting system was analysed using natural language processing. Text or word patterns were then extracted. Using the patterns, non-linear algorithms could identify variable interactions that are positively or negatively associated with an outcome variable	- Nurses comprised 89% of all incident reports - Levels 0 and 1 (incidents with no direct impact/no substantial damage to patients) accounted for 88% - Medical staff may have contributed to the safe environment by reporting good practices - Hospital staff tend to focus on individual actions rather than on a systematic approach
Anderson et al., (2020) [54]	A large health trust in London (United Kingdom)	Using Safety-II and resilient healthcare principles to learn from Never Events	*Unique tool* Four methods of analysis were used to review the Root Cause Analysis (RCA) reports: 1. A framework of analytic effectiveness was used to rate the reports on six dimensions 2. A framework of six resilience domains was used to rate the extent to which the reports incorporated resilient healthcare principles 3. A qualitative thematic analysis to determine common misalignments identified in the reports to highlight potential learning opportunities 4. The actions proposed in the reports were also assessed and rated as limited, satisfactory or comprehensive	Quantitative - Even the best RCA reports scored just over half of the total possible score - 144 misalignments were found and just under half of these were not associated with any actions Qualitative The main weaknesses in the RCA reports were: 1. Not understanding/describing work-as-done 2. Not addressing misalignments that indicate weakness in systems, such as staff shortages 3. Not considering how the identified problem could affect other areas 4. Not addressing staff well-being 5. Inadequate checking processes are usually addressed by adding items to checklists after a problem has occurred, rather than using this as an opportunity to think holistically and design a better checklist 6. Not addressing policy problems often. If a policy was not followed, few attempts were made to determine why
**ORGANISATIONAL LEARNING TOOLS: Learning through interviews/observations**				
Anderson et al., (2020) [56]	Older people´s unit and Emergency Department (United Kingdom)	Concepts for Applying Resilience Engineering (CARE)	*Unique tool* The CARE model focuses attention on misalignments between demand and capacity, staff adaptations in response to misalignments and emergent outcomes - Focus on understanding work as it is done in practice - Maintain a neutral stance about what the problems and difficulties are - Study how goals are achieved despite difficulties and understand in-depth the contextual factors that challenge workers - Use resilient healthcare concepts to look for aspects of the work system that do not support workers - Identify potential solutions that can support worker adaptation to challenges	- It is feasible to use CARE to understand work-as-done in practice, identify weak processes and propose interventions designed to strengthen adaptive capacity - A semi-structured topic guide is offered as a help to perform the interviews
Hegde et al., (2020) [62]	A large multispecialty hospital system. Health care professionals from a variety of units. (United States of America)	Knowledge Elicitation to Understand Resilience	*Unique tool* An interview protocol to elicit information from frontline clinical providers about factors that underlie resilience in everyday clinical work Interview protocol - Incident identification - Timeline and decision point identification - Deepening - ‘What if?’ queries Qualitative analysis identifies themes related to adaptation and variability in everyday clinical work	- The interview protocol helped identify resilience capabilities (monitoring, anticipating and learning) regardless of any event - The method was a shift from techniques that focus knowledge elicitation on specific events - The framework represents resilience capabilities relevant to specific issues and can be used as a trigger for deeper learning of work-as-done
Sanford et al., (2022) [57]	Two surgical wards, an older adult ward, a critical care unit, an Acute Assessment Unit (United Kingdom)	Concepts for Applying Resilience Engineering (The CARE 2.0 model)	*Unique tool* The CARE 2.0 include types of misalignments and corresponding adaptions, which can be used to better understand work-as-done Misalignment types are divided into communication, equipment, process, space, staffing and workflow. Adaptation types are divided into process, resource distribution and extra-role performance	- A table with adaptation and misalignments is offered as an extension to the CARE model - The CARE model 20.0 specifies the types of misalignments and adaptations observed in hospital teams and thus provides further guidance for understanding adaptive capacity and how it is affected in practice
**SYSTEMIC LEARNING TOOLS: Learning through performance evaluation**				
Borghini et al., (2021) [67]	10 Italian regions (Italy)	Learning from Excellence (LfE)	*Unique tool* - Identifying the best practices among 10 Italian regions that share an interregional performance evaluation system (IRPES) - Sharing experiences, management models and data across a professional community to spread the best practices model throughout the IRPES	Quantitative - Seven best-performing units were identified among the 42 units of analysis (ranking > 3.01) - The best performer’s result was 3.43, while the worst performer reached a value of 1.46 Qualitative Communication, trust and shared goals among health professionals played a key role among the best performing hospitals
**SYSTEMIC LEARNING TOOLS: Learning through asking questions in situ**				
Watt et al., (2019) [58]	Three large teaching hospitals UK (United Kingdom)	Resilience in the blood transfusion process: every-day and long-term adaptations to ‘normal’ work	*Unique tool* Real-time data collection was undertaken with employees being questioned while performing each of the steps of the transfusion process The questions were as follows: - Please give a short outline of the biggest or most recent difficulty that you faced when carrying out this procedure, and what did you do about it? (open response format) - How supportive was your manager/department for how you solved the issue? (5-point Likert scale) The Systems Engineering Initiative for Patient Safety 2.0 (SEIPS) was chosen for analysis	Triggers for adaptations were typically: - 1)Staff-related (understaffing, insufficient knowledge and training) - 2)Problems with tools and technology - As staff members are unable to solve the source of the difficulty, they are forced to adapt elsewhere in the system, within their circle of influence - An enhanced CARE framework was proposed based on the findings of triggers and types of adaptations and provided in-depth insights into work-as-done in blood transfusions - Two simple questions on adaptations of the enhanced CARE framework can provide in-depth insights into work-as-done in blood transfusion, subsequently informing a meaningful system analysis and improvement

#### Setting

The most frequent settings of the included articles describing the tools were children’s hospitals, including paediatric and neonatal critical care units and surgical wards [59–61, 65, 68, 70]. The second most frequent were surgical settings, including surgical teams, anaesthesia units, perioperative units and surgical wards [57, 63, 69, 71]. However, the tools had a wide variety in settings, including elderly units, medical units and whole hospitals. No articles described tools applied in primary care.

#### Outcome

Interventions were mostly concerned with validating the tools’ ability to provide in-depth insights into WAD and their positive effect on staff morale, positive reporting, burnout and well-being and feasibility [56–58, 62, 63]. Most of the articles used subjective measurements through staff surveys and interviews. A few used objective measures (reduced antimicrobial consumption, cardiac arrests and the number of reports regarding incidents with no impact on patients) [53, 59, 66]. The Maslach Burnout Inventory and Siegrist survey [74, 75] were used to evaluate burnout and high-effort/low-reward conditions in one study [68]. The findings per theme and sub-theme will be discussed in the following paragraphs.

#### Quality of evidence

While all the qualitative studies (*n* = 5) and most of the quantitative studies (*n* = 4) were of high methodological quality, most mixed methods studies (*n* = 6) did not adhere to the quality criteria of each tradition of the methods involved. A detailed presentation of the ratings of each quality criterion is available in supplementary file 2.

### Results of synthesis

We divided the tools in themes based on three levels of learning in healthcare: *situated-, structural- and systemic learning tools* (Table 3). The first theme, *situated learning tools*, referred to tools used by healthcare professionals at the frontline, and was divided into three sub-themes: tools based on peer reporting, positive debriefing, and safety huddles (*n* = 15). The second theme, *organisational learning tools*, referred to tools used across units in the organisation and was divided into two sub-themes: tools based on learning through incident reporting systems and interviewing and/or observations (*n* = 5). The third theme, *systemic learning tools*, referred to tools to learn on a national or regional level, and was divided into two sub-themes: tools based on learning through performance evaluation and questioning in situ (n = 2). The majority of the included articles described *situated learning tools*, of which Learning from Excellence (LfE) is the most frequently researched learning tool to operationalise the Safety-II perspective [61]. Tools to learn at a systemic level were the least frequently type presented in the included articles. Table 3 presents and describes the themes and sub-themes, and places the included articles in the three levels of healthcare.

**Table 3:** Themes and sub-themes of results

	TOOLS	DESCRIPTION	*N*	REFERENCES
**Theme**	1. **SITUATED LEARNING TOOLS **	**Locally (e.g. the sharp end of healthcare, hospital units)**	**15**	
**Sub-themes**	1.2 Learning through peer reporting	Learning from collecting and storing episodes of peer-reported excellence	6	[53, 55, 59–61, 68]
	1.3 Learning through positive debriefing	´Learning from how difficulties are overcome; adaptations that worked and simulation programs focusing on what went well´ [36]	6	[63, 64, 69–72]
	1.4 Learning through safety huddles	Learning through discussing and sharing learning in team meetings	3	[52, 65, 73]
**Theme**	2. **ORGANISATIONAL LEARNING TOOLS**	**Across the organisation (e.g. between hospital units)**	**5**	
**Sub-themes**	2.1 Learning through incident reporting systems	Identifying good practice and adaptations by analysing aggregated data at the organisational level, e.g. adverse incidents [36]	2	[54, 66]
	2.2 Learning through interviewing/observation	Organisational mechanisms for discussing and sharing and disseminating learning across the organisation [36]	3	[56, 57, 62]
**Theme**	3. **SYSTEMIC LEARNING TOOLS**	**Nationally (e.g. regions, between hospitals)**	**2**	
**Sub-themes**	3.1 Learning through performance evaluation	Learning through a regional performance evaluation system—system mechanisms for discussing and sharing learning [36]	1	[67]
	3.2 Learning through asking questions in situ	System learning from staff experiences. Learning through capturing, aggregating and analysing data, and identifying learning potentials—system mechanisms for discussing and sharing learning [36]	1	[58]

#### Situated learning tools

Situated learning tools can be used locally by healthcare professionals at the bedside and can be divided into *peer reporting, positive debriefing* or *safety huddles*.

LfE is a *peer reporting tool*, meaning that a peer reports a colleague’s excellent performance either on paper or using an electronic reporting system juxtaposed with the incident reporting system. The excellence report is read by an inter-professional team, and forwarded to the receiver. Some excellence reports are explored in depth by the use of appreciative inquiry and with attendance by recipients and reporters. This episode of excellence can be disseminated to the rest of the staff, so that ideas of new ways of doing things can be explored and implemented [61]. LfE principles can also be used at the systemic level, by identifying the best practices in several regions based on performance evaluation systems [67]. The hypothesis behind LfE is that it can “augment learning, enhance patient outcomes and experience through quality improvement work and positively impact resilience and culture in the workplace” [61]. Three of the articles evaluated the impact of LfE on staff [60, 61, 68]. They claimed that LfE has a positive effect on staff morale and positive reporting, as well as increased well-being and reduced stress. However, these three studies had unmet criteria on the Mixed Methods Appraisal Tool, which indicates that the articles were of moderate methodological quality. Two high quality studies measured a positive impact of LfE on antimicrobial consumption and cardiac arrest [53, 59]. One explored the impact of LfE on organisational performance identified as the following eight outcomes [55]: positive effects on workplace culture, motivation, morale, patient experience, patient safety, positive emotions, relationships, and resilience. The authors noted that further research is needed regarding the impact of LfE on clinical outcomes and staff learning.

*Positive debriefing tools* create an opportunity to systematically discuss episodes after delivering care to a patient, thereby improving healthcare. All six tools in this sub-theme included a structured set of open questions to use for reflection, e.g.: What went right? What helped or hindered? What can we learn from this? [71]. The reflections were used by healthcare professionals to learn from, either alone or together as a team. The articles described a wide variety of tool content, including reflecting alone in writing [63, 70], team reflection after a patient case [71], reflections at mortality and morbidity meetings [69], and debriefing during simulations [64, 72]. Three of the articles were empirical papers and evaluated the tool outcome [63, 64, 69]. They found that their positive debriefing tools added value to the debriefing, were feasible to implement and elicited narratives of successful adaptation. The authors noted that further research is needed regarding how to maintain momentum after implementing debriefing tools, how to quantify daily improvement achieved through debriefing and what is the cultural impact of embedding such debriefing in healthcare organisations.

*Safety-II- inspired safety huddles* can be described as a multidisciplinary, brief exchange of information about WAD, including work that goes well at the beginning of every shift. Subjects for discussion may include: how did you recognise changes to a situation, and how did you handle this. The huddles should take place regularly, if not daily, then at least weekly, and learning should be documented in a calendar [52]. Three articles described such a tool, and only one evaluated empirically the experiences of learning from WAD [65]: No differences in safety culture for most comparisons before and after the intervention were found. It was perceived as difficult to introduce reflections based on learning from everything that happened, including work that went well. The study had high risk of nonresponse bias. The authors suggest that further research is needed to understand how to best implement Safety-II inspired safety huddles, and to determine whether increased understanding of the purpose will improve patient safety [65]. Safety tree analysis can be used to help structure discussions on what went well in safety huddles [73]. The idea is to identify a good thing that you want to happen, and then, in turn, identify the pre-conditions for it to happen, etc.

#### Organisational learning tools

Organisational learning tools can be used to learn across an organisation and divided into *incident reporting systems* and *interviews and/or observations*.

Two articles approached* incident reporting systems,* which are traditionally used to capture patient harm, with a Safety-II view [54, 66]. Both are empirical studies; however, they differ widely in content and results measured. The mixed method study used a framework of analytic effectiveness to determine common misalignments identified in the root cause analysis of Never Events [54]. The study found that even the best root cause analysis was inefficient, and half of the misalignments were not associated with any actions. The authors conclude that incorporating Safety-II approach in safety thinking is necessary to improve the quality of care, and they provide a number of ways root cause analysis can be improved. By using Never Events as a window on the work system and by applying concepts from resilient healthcare that focus on WAD and adaptive capacity, opportunities to identify vulnerabilities and strengthen systems can be found. The quantitative study extracted data from the incident reporting system and utilised statistical text analysis to identify common themes behind good practices, improved quality and safety based on Safety-II principles [66]. The study found that nurses comprised 88% of the incident reports, and incidents with no direct impact or no substantial impact on patients accounted for 88% of the reports. By constantly reporting good practices, nurses and other healthcare professionals may have contributed to patient safety. However, healthcare professionals tend to focus on individual actions rather than a systemic approach.

Three qualitative studies described tools that used *interviews and/or observations* to help understand WAD with the aim to proactively identify system vulnerabilities and propose quality interventions to strengthen adaptive capacity [56, 57, 62]. The Concepts for Applying Resilience Engineering (CARE) model was used in two of the studies [56, 57]. The CARE model proposes that adaptivity is characterised by misalignments between demand and capacity, as it is impossible to perfectly align capacity to meet constantly changing demands, such as patient emergency etc. The researchers collected data based on non-participant observations and ethnographic interviews [56, 57], followed by semi-structured interviews [56]. This was time consuming; the observation sessions alone totalled between 60 and 104 h, and the fifteen interviews lasted between 45–90 min each. Then, deductive-inductive thematic analysis of the data commenced. A semistructured topic guide was offered as help to perform interviews [56]. CARE was feasible to obtain in-depth knowledge of WAD, and helped identify weak processes and propose interventions. An extension of the CARE model was proposed, to explicitly specify the misalignments and adaptations observed in hospital teams (CARE 2.0) [57]. As adaptations to work do not only occur because of misalignments, further research is needed to explore other factors that could precipitate adaptations and the outcomes of these [57].

The third study aimed to learn how healthcare professionals achieve safe care regardless of any events using an interview protocol that was offered as a tool [62]. The interview may begin with “Tell me about a time when you dealt with a complex patient case” ([62] p. 77). The researchers used thematic analysis to analyse the data. The authors concluded that the protocol helped them understand what went right in everyday work, regardless of any events. The findings were used to develop a Resilience Mapping Framework, which illustrates the four capabilities (monitor, anticipate, respond, learn) and their relationships across different levels of the organisational scale (individual at the frontline, unit, department, institution and industry). The Resilience Mapping Framework can be utilised to proactively investigate ways to support or enhance system resilience.

#### Systemic learning tools

Systemic learning tools are used nationally or between hospitals, and can be divided into *performance evaluation* and *questioning *in situ.

The mixed method study used an interregional *performance evaluation* system to evaluate whether LfE could be an effective method to identify and spread best practices among 10 Italian healthcare regions [67]. The authors concluded that it had the potential to promote improvement processes and boost personnel resilience and the organisational working climate. However, how health professionals can learn from positive results should be further investigated.

Real-time data collection based on *asking healthcare professionals questions *in situ*,* while they perform normal blood transfusion processes, was undertaken across three large hospitals, to understand what individuals normally do when things go wrong [58]. In this qualitative study, two questions were asked and offered as a tool: (1) *Give a short outline of the biggest most recent difficulty you have encountered and what did you do about it?* (2) *How supportive was your manager of how you solved it?* These questions informed the researcher that the most typical triggers for staff-related adaptations were problems with technology. As healthcare professionals often are unable to solve technological problems, they are forced to adapt elsewhere within their circle of control. This tool can be used nationwide in a vein-to-vein audit of all transfusion processes.

## Discussion

This systematic review aimed to provide a qualitative overview of practical tools to learn from success or work-as-done in healthcare practices, using a mixed studies systematic review. Furthermore, it assessed the tools regarding their feasibility, acceptability, and effectiveness. It was found that tools to learn from success or WAD can be categorised according to the three different levels of healthcare where they impact: the situated, structural and systemic levels.

Situated learning tools, i.e. tools used by healthcare professionals on the frontline of healthcare, consist of tools based on peer reporting, positive debriefing, and safety huddles. Both peer reporting (i.e. LfE) and positive debriefing tools are deemed feasible to implement and easy to understand (reporting and learning from episodes of good practice). They add value both to healthcare professionals` well-being and in providing a deeper understanding of ways to keep patients safe in a complex and ever-changing conditions.

The Safety-II focused safety-huddle tool `Green Line`, however, was not feasible to implement [65]. The frontline healthcare professionals did not understand the underpinning concepts of Safety-II and found it hard to learn from situations that had been resolved, as these experiences were taken for granted [65]. Verbally expressing tacit knowledge, such as in every-day WAD, is known to be difficult [36]. Although the Safety-II paradigm has been discussed theoretically by researchers for years, healthcare professionals are highly unfamiliar with the concept of resilience as a perspective for WAD, quality of care, and patient safety [76]. Indeed, critiques have argued that the discourse around resilience is “fleeting, ambiguous and disconnected from operational reality” ([77] p.7). A lack of practical guidance on how to operationalise the key concepts of Safety-II, such as learning from everyday work, has hampered Safety-II´s adoption in healthcare [33]. A pre-requisite for implementing Safety-II in healthcare is to translate the resilience concept into meaningful practical concepts. Such tools have started to emerge, such as the serious videogame Resilience Challenge and the Resilience in Healthcare tool [76, 78]. The latter is found to successfully introduce the resilience perspective to healthcare professionals by developing shared reflection, understanding, focus and language [76].

Since the Safety-II perspective is in its infancy in healthcare, it is surprising to find as many as 16 tools, most of which have been published in the last 3 years. The tools have different learning focuses, but none of them stand out as being better than the others. A variety of tools will likely be developed in the forthcoming years, and contribute to increased knowledge regarding what types of tools work for whom [79]. Learning from everyday work is fundamental in Safety-II, however, no guidance on how to develop such learning tools or go about this process of learning has been found [24, 80]. As learning does not just happen, which unfortunately is true for most learning systems, it is important to have a theoretical anchoring for developing future resilience learning tools [8, 9, 79]. Finally, a significant study on what constitutes good tools to learn from success and WAD has arrived [79]. Its key elements are: using a collaborative approach, having high flexibility and usability and creating spaces for reflection where examples of good practice can be shared. Most of the tools found in this review have several of these features. Creating spaces for inter-professional reflection is challenging for most tools, apart from debriefing tools, as, for example, few physicians have designated time to allow continuous involvement [81]. However, inter-professional reflection is essential for enabling quality improvements and developing a shared understanding [82–85]. In the future, this may be the hardest challenge to overcome, as well as to translate resilience into meaningful practical understanding [77].

Organisational learning tools, i.e. tools used across units in the organisation, consist of tools based on learning through incident reporting systems, and learning through interviews and observations. This is a time-consuming endeavour, and requires skilled researchers. A learning tool for including Safety-II perspectives in the root cause analysis of Never Events can complement existing root cause analysis investigations [54]. This is important, as opportunities to create safer systems are lost from many root cause analysis reports, as can be seen by the continued occurrence of Never Events [86]. In addition, incident reporting systems have been underutilised [87]. Tools to look at incidents with a Safety-II lens through text mining are exciting and may be developed further to complement existing incident reporting systems [66].

Interestingly, organisational tools, as well as systemic tools, used interviews and observations as strategies to obtain in-depth information about WAD in practice. Tools such as CARE focus on understanding work as it is done by healthcare professionals and, by taking a neutral stance, studying how the goals are achieved despite difficulties [56]. This in-depth understanding is the crux of Safety-II, as it mirrors the complexity of work, and not the linear WAI [15]. This may identify potential solutions to better support worker adaptation and improve patient safety [56, 57, 62]. This sharing and disseminating learning across the organisation, is a breath of fresh air in patient safety practices, and something we hope to see much more of in the future [36, 85].

*Systemic learning tools* refer to tools on a national level, based on learning through performance evaluation, and learning through questioning and observation. By identifying the best practices in several regions based on performance evaluation systems, principles for LfE can also be used at the systemic level. This strategy was implemented in Italy, with healthcare professionals and managers from different regions coming together to share experiences of good performance [67]. In addition, albeit with mostly narrative value, this is an example of collaborative learning across different levels and contexts and precisely what a systems perspective demands [85]. Another good example of systemic learning is observing and questioning healthcare professionals during the transfusion process, which still causes a handful of deaths per year [58]. This tool provided a deeper understanding of what triggers adaptations and looked for system-related causes of adverse events. As healthcare professionals are often unable to resolve the source of the problem, they are forced to adapt elsewhere in the transfusion process, -i.e. within their circle of control. Therefore, this systems approach to safety certainly has an advantage over blaming individuals, as is often the case with traditional analysis of incidents [87].

### Study limitations

As described in the methods chapter, it was decided (during the research process) to exclude FRAM, appreciative inquiry and positive deviance tools, which others may have found natural to include in this systematic review. This was done for pragmatic reasons, as systematic reviews of studies with these tools had recently been done. In addition, appreciative inquiry and positive deviance came before the Safety-II paradigm, and the reviewers wanted to find tools that operationalised the Safety-II paradigm. There is also the case of exclusion/inclusion bias that cannot be ignored, although mitigated by using the aforementioned techniques. The inclusion of non-empirical papers may be both a limitation and a strength of the result.

## Conclusions

This systematic review shows the emergence of a growing number of tools to learn from success and WAD at all levels of healthcare. The tools may help understand, and learn from the constant adaptations done by healthcare professionals every day to keep patients safe.

The review shows a variety in the content of the tools and in the measured outcomes. All of the articles came from hospitals in high-income countries; none are from studies in primary care. Few studies focused on patient outcomes. We suggest that future research focuses on measuring patient safety outcomes and exploring how this is impacted by learning tools, as well as whether increased understanding and application of the Safety-II concept improves patient safety. Learning in healthcare is not easy, as healthcare is a complex non-linear system [88]. Further development of practical tools is needed to learn from both errors and success, to improve patient safety [33].

## Supplementary Information

Supplementary Material 1.

Supplementary Material 2.

## Data Availability

All data generated or analysed during this study are included in this published article [and its supplementary information files].

## Abbreviations

CARE: The Concepts for Applying Resilience Engineering
FRAM: Functional Resonance Analysis Method
LfE: Learning from excellence
PRISMA: Preferred Reporting Items for Systematic Reviews and Meta-Analyses
WAD: Work-as-done
WAI: Work-as-imagined
