# Supplementary material for: Strategies and tools to learn from work that goes well within healthcare patient safety practices: a mixed methods systematic review
Source: BMC Health Serv Res. 2025 Apr 14;25:538. doi: 10.1186/s12913-025-12680-2 (PMC11995654; doi:10.1186/s12913-025-12680-2)
Supplement: Supplementary file 1 — Supplementary Material 1. [file 12913_2025_12680_MOESM1_ESM.docx]

**Supplementary file 1.**

Strategies and Tools to Learn from Work that Goes Well within Healthcare Patient Safety Practices. A Mixed Methods Systematic Review

**Search strategies**

Date searched: 17 February 2023

Total number of hits: 15 259

Total number of hits after removing duplidates: 5 468

Database: **Ovid MEDLINE ALL**1946 to February 16, 2023

| # | Searches | Results |
| --- | --- | --- |
| 1 | "safety II".mp. | 111 |
| 2 | "safety 2".mp. | 225 |
| 3 | "resilien* engineer*".mp. | 95 |
| 4 | or/1-3 | 417 |
| 5 | "Delivery of Health Care"/ or exp Health Personnel/ or exp Hospitals/ | 968095 |
| 6 | ("health care" or healthcare or ((health or medical) adj (personnel or professional* or worker* or provider* or staff)) or (health and staff) or hospital* or nurse* or nursing or physician* or doctor* or clinician*).ti,ab,kf. | 3172674 |
| 7 | or/5-6 | 3541347 |
| 8 | Learning/ | 78910 |
| 9 | learn*.ti,ab,kf. | 579499 |
| 10 | or/8-9 | 595130 |
| 11 | Resilience, Psychological/ | 8177 |
| 12 | resilien*.ti,ab,kf. | 55831 |
| 13 | ("work as done" or "work as imagined" or "everyday work" or "successful work" or (positive adj (devian* or reporting or feedback* or event*)) or "adaptive capacit*" or appreciati* or "what goes well" or "what went well" or "what goes right" or "what went right" or "work goes well" or "work went well" or "work goes right" or "work went right" or "what we do well" or "things that go right" or "things go right").ti,ab,kf. | 47091 |
| 14 | or/11-13 | 103453 |
| 15 | (learn* adj6 (excellence or success$2)).ti,ab,kf. | 3405 |
| 16 | Patient safety/ or Safety management/ or Risk management/ or Medical Errors/ or Patient Harm/ or Quality of Health Care/ or Quality improvement/ | 173818 |
| 17 | (safety or harm or risk* or error* or incident* or (adverse adj3 event*) or quality or improv* or enhanc*).ti,ab,kf. | 8328914 |
| 18 | or/16-17 | 8385233 |
| 19 | 4 or (7 and 10 and 14 and 18) or (7 and 15 and 18) | 2650 |

Database: **Embase (Ovid)**1974 to 2023 February 16

| # | Searches | Results |
| --- | --- | --- |
| 1 | "safety II".mp. | 174 |
| 2 | "safety 2".mp. | 462 |
| 3 | "resilien* engineer*".mp. | 154 |
| 4 | or/1-3 | 763 |
| 5 | health care delivery/ or exp health care personnel/ or exp hospital/ | 3108631 |
| 6 | ("health care" or healthcare or ((health or medical) adj (personnel or professional* or worker* or provider* or staff)) or (health and staff) or hospital* or nurse* or nursing or physician* or doctor* or clinician*).ti,ab,kf. | 4448532 |
| 7 | or/5-6 | 5823119 |
| 8 | learning/ | 242747 |
| 9 | learn*.ti,ab,kf. | 740162 |
| 10 | or/8-9 | 783878 |
| 11 | psychological resilience/ | 8131 |
| 12 | resilien*.ti,ab,kf. | 62326 |
| 13 | ("work as done" or "work as imagined" or "everyday work" or "successful work" or (positive adj (devian* or reporting or feedback* or event*)) or adaptive capacit* or appreciati* or "what goes well" or "what went well" or "what goes right" or "what went right" or "work goes well" or "work went well" or "work goes right" or "work went right" or "what we do well" or "things that go right" or "things go right").ti,ab,kf. | 60286 |
| 14 | or/11-13 | 123275 |
| 15 | (learn* adj6 (excellence or success$2)).ti,ab,kf. | 4101 |
| 16 | patient safety/ or risk management/ or medical error/ or patient harm/ or patient risk/ or health care quality/ | 466333 |
| 17 | (safety or harm or risk* or error* or incident* or (adverse adj3 event*) or quality or improv* or enhanc*).ti,ab,kf. | 11313698 |
| 18 | or/16-17 | 11486139 |
| 19 | 4 or (7 and 10 and 14 and 18) or (7 and 15 and 18) | 5165 |
| 20 | limit 19 to conference abstracts | 2143 |
| 21 | 19 not 20 | 3022 |

Database: **APA PsycInfo (Ovid)**1806 to February Week 1 2023

| # | Searches | Results |
| --- | --- | --- |
| 1 | "safety II".mp. | 30 |
| 2 | "safety 2".mp. | 101 |
| 3 | "resilien* engineer*".mp. | 95 |
| 4 | or/1-3 | 220 |
| 5 | health care delivery/ or exp health personnel/ or exp hospitals/ | 224577 |
| 6 | ("health care" or healthcare or ((health or medical) adj (personnel or professional* or worker* or provider* or staff)) or (health and staff) or hospital* or nurse* or nursing or physician* or doctor* or clinician*).ti,ab,id. | 592030 |
| 7 | or/5-6 | 672519 |
| 8 | learning/ | 75939 |
| 9 | learn*.ti,ab,id. | 542831 |
| 10 | or/8-9 | 546790 |
| 11 | "resilience (psychological)"/ | 20008 |
| 12 | resilien*.ti,ab,id. | 42527 |
| 13 | ("work as done" or "work as imagined" or "everyday work" or "successful work" or (positive adj (devian* or reporting or feedback* or event*)) or "adaptive capacit*" or appreciati* or "what goes well" or "what went well" or "what goes right" or "what went right" or "work goes well" or "work went well" or "work goes right" or "work went right" or "what we do well" or "things that go right" or "things go right").ti,ab,id. | 29677 |
| 14 | or/11-13 | 72434 |
| 15 | (learn* adj6 (excellence or success$2)).ti,ab,id. | 4967 |
| 16 | patient safety/ or risk management/ or "quality of care"/ | 23573 |
| 17 | (safety or harm or risk* or error* or incident* or (adverse adj3 event*) or quality or improv* or enhanc*).ti,ab,id. | 1461055 |
| 18 | or/16-17 | 1462647 |
| 19 | 4 or (7 and 10 and 14 and 18) or (7 and 15 and 18) | 1055 |

Database: **Cinahl (Ebsco)**

| # | Query | Results |
| --- | --- | --- |
| S1 | TX "safety II" | 47 |
| S2 | TX "safety 2" | 153 |
| S3 | TX "resilien* engineer*" | 41 |
| S4 | S1 OR S2 OR S3 | 238 |
| S5 | MH ("Health Care Delivery" or "Health Personnel+" or "Hospitals+") | 776,745 |
| S6 | TI ( "health care" or healthcare or ((health or medical) N0 (personnel or professional* or worker* or provider* or staff)) or (health and staff) or hospital* or nurse* or nursing or physician* or doctor* or clinician* ) OR AB ( "health care" or healthcare or ((health or medical) N0 (personnel or professional* or worker* or provider* or staff)) or (health and staff) or hospital* or nurse* or nursing or physician* or doctor* or clinician* ) | 1,612,272 |
| S7 | S5 OR S6 | 1,997,237 |
| S8 | (MH "Learning") | 27,598 |
| S9 | TI learn* OR AB learn* | 209,239 |
| S10 | S8 OR S9 | 219,552 |
| S11 | TI resilien* OR AB resilien* | 22,117 |
| S12 | TI ( "work as done" or "work as imagined" or "everyday work" or "successful work" or (positive N0 (devian* or reporting or feedback* or event*)) or "adapdive capacit*" or appreciati* or "what goes well" or "what went well" or "what goes right" or "what went right" or "work goes well" or "work went well" or "work goes right" or "work went right" or "what we do well" or "things that go right" or "things go right" ) OR AB ( "work as done" or "work as imagined" or "everyday work" or "successful work" or (positive N0 (devian* or reporting or feedback* or event*)) or "adapdive capacit*" or appreciati* or "what goes well" or "what went well" or "what goes right" or "what went right" or "work goes well" or "work went well" or "work goes right" or "work went right" or "what we do well" or "things that go right" or "things go right" ) | 13,717 |
| S13 | S11 OR S12 | 35,636 |
| S14 | TI ( learn* N5 (excellence or success*) ) OR AB ( learn* N5 (excellence or success*) ) | 3,285 |
| S15 | MH ("Patient Safety" OR "Adverse Health Care Event" OR "Quality of Health Care" OR "Quality improvement") | 209,060 |
| S16 | TI ( safety or harm or risk* or error* or incident* (adverse N2 event*) or quality or improv* or enhanc* ) OR AB ( safety or harm or risk* or error* or incident* (adverse N2 event*) or quality or improv* or enhanc* ) | 2,142,324 |
| S17 | S15 OR S16 | 2,229,487 |
| S18 | S4 OR (S7 AND S10 AND S13 AND S17) OR (S7 AND S14 AND S17) | 2,135 |

Database: **Cochrane Central Register of Controlled Trials (Wiley)**

| **ID** | **Search** | **Hits** |
| --- | --- | --- |
| #1 | ("safety II" or "safety 2"):ti,ab,kw | 166 |
| #2 | (resilien* NEXT engineer*):ti,ab,kw | 0 |
| #3 | MeSH descriptor: [Delivery of Health Care] this term only | 1314 |
| #4 | MeSH descriptor: [Health Personnel] explode all trees | 12964 |
| #5 | MeSH descriptor: [Hospitals] explode all trees | 4835 |
| #6 | ("health care" or healthcare or ((health or medical) NEXT (personnel or professional* or worker* or provider* or staff)) or (health and staff) or hospital* or nurse* or nursing or physician* or doctor* or clinician*):ti,ab,kw | 372280 |
| #7 | #3 OR #4 OR #5 OR #6 | 374714 |
| #8 | MeSH descriptor: [Learning] this term only | 3060 |
| #9 | (learn*):ti,ab,kw | 42057 |
| #10 | #8 OR #9 | 42057 |
| #11 | MeSH descriptor: [Resilience, Psychological] this term only | 376 |
| #12 | (resilien*):ti,ab,kw | 3530 |
| #13 | ("work as done" or "work as imagined" or "everyday work" or "successful work" or (positive NEXT (devian* or reporting or feedback* or event*)) or "adaptive capacit*" or appreciati* or "what goes well" or "what went well" or "what goes right" or "what went right" or "work goes well" or "work went well" or "work goes right" or "work went right" or "what we do well" or "things that go right" or "things go right"):ti,ab,kw | 1712 |
| #14 | #11 OR #12 OR #13 | 5213 |
| #15 | (learn* NEAR/5 (excellence or success*)):ti,ab,kw | 462 |
| #16 | MeSH descriptor: [Patient Safety] this term only | 849 |
| #17 | MeSH descriptor: [Safety Management] this term only | 208 |
| #18 | MeSH descriptor: [Risk Management] this term only | 181 |
| #19 | MeSH descriptor: [Medical Errors] this term only | 193 |
| #20 | MeSH descriptor: [Patient Harm] this term only | 6 |
| #21 | MeSH descriptor: [Quality of Health Care] this term only | 1148 |
| #22 | MeSH descriptor: [Quality Improvement] this term only | 1004 |
| #23 | (safety or harm or risk* or error* or incident* or (adverse NEAR/2 event*) or quality or improv* or enhanc*):ti,ab,kw | 991004 |
| #24 | #16 OR #17 OR #18 OR #19 OR #20 OR #21 OR #22 OR #23 | 991004 |
| #25 | #1 OR #2 OR (#7 AND #10 AND #14 AND #24) OR (#7 AND #15 AND #24) | 447 |
| #26 | #1 OR #2 OR (#7 AND #10 AND #14 AND #24) OR (#7 AND #15 AND #24) in Trials | 439 |

Database: **Web of Science (Clarivate)
Science Citation Index, Social Sciences Citation Index, Arts & Humanities, Emerging Sources Citation Index**

| **#** | **Query** | **Hits** |
| --- | --- | --- |
| #1 | TS=("safety II" OR "safety 2") | 471 |
| #2 | TS=("resilien* engineer*") | 382 |
| #3 | TS=("health care" OR healthcare OR ((health OR medical) NEAR/0 (personnel OR professional* OR worker* OR provider* OR staff)) OR (health and staff) OR hospital* OR nurse* OR nursing OR physician* OR doctor* OR clinician*) | 2,714,999 |
| #4 | TS=learn* | 1,258,164 |
| #5 | TS=resilien* | 158,527 |
| #6 | TS=("work as done" OR "work as imagined" OR "everyday work" OR "successful work" OR "positive devian*" OR "positive reporting" OR "positive feedback*" OR "positive event*" OR "adaptive capacit*" OR appreciati* OR "what goes well" OR "what went well" OR "what goes right" OR "what went right" OR "work goes well" OR "work went well" OR "work goes right" OR "work went right" OR "what we do well" OR "things that go right" OR "things go right") | 76,745 |
| #7 | #5 OR #6 | 231,715 |
| #8 | TS=(learn* NEAR/5 (excellence OR success OR successes)) | 7,972 |
| #9 | TS=(safety or harm or risk* or error* or incident* (adverse NEAR/2 event*) or quality or improv* or enhanc*) | 13,738,930 |
| #10 | #1 OR #2 OR (#3 AND #4 AND #7 AND #9) OR (#3 AND #8 AND #9) | 2903 |

Database: **Scopus (Elsevier)**

| **#** | **Query** | **Hits** |
| --- | --- | --- |
| #1 | TITLE-ABS-KEY ( {safety ii} OR {safety 2}) | 129 |
| #2 | TITLE-ABS-KEY ( "resilien* engineer*" ) | 839 |
| #3 | TITLE-ABS ( "health care" OR healthcare OR ( ( health OR medical ) PRE/0 ( personnel OR professional* OR worker* OR provider* OR staff ) ) OR ( health AND staff ) OR hospital* OR nurse* OR nursing OR physician* OR doctor* OR clinician* ) OR AUTHKEY ( "health care" OR healthcare OR ( ( health OR medical ) PRE/0 ( personnel OR professional* OR worker* OR provider* OR staff ) ) OR ( health AND staff ) OR hospital* OR nurse* OR nursing OR physician* OR doctor* OR clinician* ) | 4,139,361 |
| #4 | TITLE-ABS ( learn* ) OR AUTHKEY ( learn* ) | 2,524,024 |
| #5 | TITLE-ABS ( resilien* OR {work as done} OR {work as imagined} OR {everyday work} OR {successful work} OR ( positive PRE/0 ( devian* OR reporting OR feedback* OR event* ) ) OR {adaptive capacit*} OR appreciati* OR {what goes well} OR {what went well} OR {what goes right} OR {what went right} OR {work goes well} OR {work went well} OR {work goes right} OR {work went right} OR {what we do well} OR {things that go right} OR {things go right} ) OR AUTHKEY ( resilien* OR {work as done} OR {work as imagined} OR {everyday work} OR {successful work} OR ( positive PRE/0 ( devian* OR reporting OR feedback* OR event* ) ) OR {adaptive capacit*} OR appreciati* OR {what goes well} OR {what went well} OR {what goes right} OR {what went right} OR {work goes well} OR {work went well} OR {work goes right} OR {work went right} OR {what we do well} OR {things that go right} OR {things go right} ) | 329,073 |
| #6 | TITLE-ABS ( learn* PRE/5 ( excellence OR success OR successes ) ) OR AUTHKEY ( learn* PRE/5 ( excellence OR success OR successes ) ) | 9,688 |
| #7 | TITLE-ABS (safety or harm or risk* or error* or incident* or (adverse PRE/2 event*) or quality or improv* or enhanc*) OR AUTHKEY (safety or harm or risk* or error* or incident* or (adverse PRE/2 event*) or quality or improv* or enhanc*) | 20,429,774 |
| #8 | #1 OR #2 OR (#3 AND #4 AND #5 AND #7) OR (#3 AND #6 AND #7) | 3,417 |
| #9 | #1 OR #2 OR (#3 AND #4 AND #5 AND #7) OR (#3 AND #6 AND #7) AND ( EXCLUDE ( DOCTYPE , "cp" ) ) | 2,955 |

**Google Scholar**

| Search string | [healthcare\|"health care"\|medical\|hospital\|hospitals learn\|learning "safety II"\|resilience\|resilient\|success\|successes\|excellence "patient safety"\|quality](https://emea01.safelinks.protection.outlook.com/?url=https%3A%2F%2Fscholar.google.com%2Fscholar%3Fhl%3Dno%26as_sdt%3D0%252C5%26q%3Dhealthcare%257C%2522health%2Bcare%2522%257Cmedical%257Chospital%257Chospitals%2Blearn%257Clearning%2B%2522safety%2BII%2522%257Cresilience%257Cresilient%257Csuccess%257Csuccesses%257Cexcellence%2B%2522patient%2Bsafety%2522%257Cquality%26btnG%3D&data=05%7C01%7C%7C646878d0e50643be4e8d08db10ce48d4%7C84df9e7fe9f640afb435aaaaaaaaaaaa%7C1%7C0%7C638122250415135938%7CUnknown%7CTWFpbGZsb3d8eyJWIjoiMC4wLjAwMDAiLCJQIjoiV2luMzIiLCJBTiI6Ik1haWwiLCJXVCI6Mn0%3D%7C3000%7C%7C%7C&sdata=l58z1yL2RLfFEkas%2FVsgCwNpKb34lNAYDO3x3qOIvbk%3D&reserved=0) |
| --- | --- |
| Results | 100 (the first 100 of about 2,8 million hits) |
