# Supplementary material for: Strategies and tools to learn from work that goes well within healthcare patient safety practices: a mixed methods systematic review
Source: BMC Health Serv Res. 2025 Apr 14;25:538. doi: 10.1186/s12913-025-12680-2 (PMC11995654; doi:10.1186/s12913-025-12680-2)
Supplement: Supplementary file 2 — Supplementary Material 2. [file 12913_2025_12680_MOESM2_ESM.docx]

**Supplementary file 2.** MMAT ratings for qualitative, quantitative, and mixed method studies

|  |  | **Qualitative studies** | | | | | **Quantitative studies** | | | | |
| --- | --- | --- | --- | --- | --- | --- | --- | --- | --- | --- | --- |
| **Category of study designs** | **Methodological quality criteria** | **Anderson et al., (2020) [56]** | **Hegde et al., (2020) [63]** | **Hegde et al., (2020) [62]** | **Sanford et al., (2022) [57]** | **Watt et al., (2019) [58]** | **Abe et al., (2022) [66]** | **Breinig et al., (2022) [68]** | **Chain et al., (2018) [60]** | **Jones et al., (2019) [59]** | **McGregor et al., (2017) [53]** |
| Screening questions  (for all types) | S1. Are there clear research questions? | Y | Y | Y | Y | Y | Y | Y | Y | Y | Y |
|  | S2. Do the collected data allow to address the research questions? | Y | Y | Y | Y | Y | Y | Y | Y | Y | Y |
| 1. Qualitative | 1.1. Is the qualitative approach appropriate to answer the research question? | Y | Y | Y | Y | Y |  |  |  |  |  |
|  | 1.2. Are the qualitative data collection methods adequate to address the research question? | Y | Y | Y | Y | Y |  |  |  |  |  |
|  | 1.3. Are the findings adequately derived from the data? | Y | Y | Y | Y | Y |  |  |  |  |  |
|  | 1.4. Is the interpretation of results sufficiently substantiated by data? | Y | Y | Y | Y | Y |  |  |  |  |  |
|  | 1.5. Is there coherence between qualitative data sources, collection, analysis and interpretation? | Y | Y | Y | Y | Y |  |  |  |  |  |
| 2. Quantitative descriptive | 2.1. Is the sampling strategy relevant to address the research question? |  |  |  |  |  | Y | Y | Y | Y | Y |
|  | 2.2. Is the sample representative of the target population? |  |  |  |  |  | Y | Y | Y | Y | Y |
|  | 2.3. Are the measurements appropriate? |  |  |  |  |  | Y | Y | CT | Y | Y |
|  | 2.4. Is the risk of nonresponse bias low? |  |  |  |  |  | Y | N | Y | Y | Y |
|  | 2.5. Is the statistical analysis appropriate to answer the research question? |  |  |  |  |  | Y | Y | CT | Y | Y |
| 3. Mixed methods | 3.1. Is there an adequate rationale for using a mixed methods design to address the research question? |  |  |  |  |  |  |  |  |  |  |
|  | 3.2. Are the different components of the study effectively integrated to answer the research question? |  |  |  |  |  |  |  |  |  |  |
|  | 3.3. Are the outputs of the integration of qualitative and quantitative components adequately interpreted? |  |  |  |  |  |  |  |  |  |  |
|  | 3.4. Are divergences and inconsistencies between quantitative and qualitative results adequately addressed? |  |  |  |  |  |  |  |  |  |  |
|  | 3.5. Do the different components of the study adhere to the quality criteria of each tradition of the methods involved? |  |  |  |  |  |  |  |  |  |  |

Y=Yes, N=No, CT=Can’t tell

|  |  | **Mixed method studies** | | | | | | |
| --- | --- | --- | --- | --- | --- | --- | --- | --- |
| **Category of study designs** | **Methodological quality criteria** | **Anderson et al., (2020) [54]** | **Bentley et al., 2021 [64]** | **Borghini et al., (2021) [67]** | **Kelly et al., (2016) [61]** | **Kletter et al., (2020) [55]** | **Verhagen et al., (2020) [69]** | **Wahl et al., (2022) [65]** |
| Screening questions  (for all types) | S1. Are there clear research questions? | Y | Y | Y | Y | Y | Y | Y |
|  | S2. Do the collected data allow to address the research questions? | Y | Y | Y | Y | Y | Y | Y |
| 1. Qualitative | 1.1. Is the qualitative approach appropriate to answer the research question? |  |  |  |  |  |  |  |
|  | 1.2. Are the qualitative data collection methods adequate to address the research question? |  |  |  |  |  |  |  |
|  | 1.3. Are the findings adequately derived from the data? |  |  |  |  |  |  |  |
|  | 1.4. Is the interpretation of results sufficiently substantiated by data? |  |  |  |  |  |  |  |
|  | 1.5. Is there coherence between qualitative data sources, collection, analysis and interpretation? |  |  |  |  |  |  |  |
| 2. Quantitative descriptive | 2.1. Is the sampling strategy relevant to address the research question? |  |  |  |  |  |  |  |
|  | 2.2. Is the sample representative of the target population? |  |  |  |  |  |  |  |
|  | 2.3. Are the measurements appropriate? |  |  |  |  |  |  |  |
|  | 2.4. Is the risk of nonresponse bias low? |  |  |  |  |  |  |  |
|  | 2.5. Is the statistical analysis appropriate to answer the research question? |  |  |  |  |  |  |  |
| 3. Mixed methods | 3.1. Is there an adequate rationale for using a mixed methods design to address the research question? | Y | Y | Y | CT | Y | CT | Y |
|  | 3.2. Are the different components of the study effectively integrated to answer the research question? | Y | CT | Y | Y | Y | Y | Y |
|  | 3.3. Are the outputs of the integration of qualitative and quantitative components adequately interpreted? | Y | Y | Y | CT | Y | CT | Y |
|  | 3.4. Are divergences and inconsistencies between quantitative and qualitative results adequately addressed? | Y | Y | Y | Y | Y | Y | Y |
|  | 3.5. Do the different components of the study adhere to the quality criteria of each tradition of the methods involved? | Y | N | CT | CT | N | CT | N |

Y=Yes, N=No, CT=Can’t tell
